# A clinical study of metastasized rectal cancer treatment: assessing a multimodal approach

**DOI:** 10.1007/s12032-014-0839-1

**Published:** 2014-01-30

**Authors:** Michaela Jung, Annica Holmqvist, Xiao-Feng Sun, Maria Albertsson

**Affiliations:** Department of Oncology, Institute of Clinical and Experimental Medicine, County Council of Östergötland, Linköping University, 58185 Linköping, Sweden

**Keywords:** Rectal cancer, Metastasis, Bevacizumab, Chemotherapy

## Abstract

Metastasized rectal cancer has long been considered incurable. During recent years, the treatment of rectal cancer patients has been improved, and nowadays, a subgroup of patients might even be cured. The aim of this study was to investigate the optimal timing of treatment in a multimodal therapy schedule in order to see whether the addition of bevacizumab (Avastin) to conventional chemotherapy was effective. The study included 39 patients with metastatic rectal cancer between 2009 and 2011, and three were excluded due to the lack of metastases or lack of follow-up information. The remaining 36 patients were divided into groups by treatment intention. The group with curative intention received mainly oxaliplatin (Eloxatin) in combination with capecitabine (Xeloda) with or without bevacizumab (Avastin) for 2 months followed by preoperative radiotherapy (RT) and surgery. Palliative patients had very different treatments depending on their needs of palliation. The median survival time for patients with curative intention was 31 months and for the palliative patients 12 months. Four of the patients (11 %) with curative intention were considered cured at the end of follow-up. The response to chemotherapy after 2-month treatment is a good prognostic sign for which patients can be cured. Long-lasting palliation can be obtained with this treatment schedule. The main side effects were gastrointestinal events, including bowel perforation, neuropathy, thrombo-embolic disease and reduced general condition. All side effects are known, and the treatment is considered tolerable. We conclude that a good treatment schedule would be oxaliplatin (Eloxatin) in combination with capecitabine (Xeloda) with or without bevacizumab (Avastin) for 2 months, followed by preoperative RT and surgery.

## Introduction

Colorectal cancer (CRC) is one of the most common malignant diseases in the world. In Sweden, there are around 5,500 new cases each year, where 2,000 of these cases are rectal cancers. Even though the local recurrence rate has been reduced for the rectal cancer patients, the mortality rate is still high, and at the time of diagnosis, around 25 % of the patients have already developed distant metastasis. Metastasized rectal cancer has long been considered incurable, and the treatment has focused on prolonging the overall survival and improving the quality of life [1]. During the recent years, the treatment modalities have improved by the use of new treatment agents, and nowadays, a subgroup with metastatic rectal cancer disease may even be possible to be cured [2].

Most studies investigate CRC as an entity, and rectal cancer as an own subgroup has only been explored to a minor extent. The main obstacle for comparing the rectal cancer group with the colon cancer group is the differences in the preoperative local treatment. For rectal cancer patients, preoperative radiotherapy (RT) in combination with surgery is essential, where colon cancer patients most commonly are treated with surgery.

The recent years researchers have started to focus on improving the time schedule between the treatments in order to avoid any delays. Multidisciplinary team (MDT) conferences where oncologists, radiologists, surgeons and pathologists meet are important tools in the treatment of CRC patients because it facilitates and coordinates the examinations and treatments [3]. It also improves the knowledge of the conference members since doctors with different modalities can exchange their thoughts.

The aim of this study was to investigate the optimal timing of treatment in a multimodal therapy schedule in order to see whether the addition of bevacizumab (Avastin) to conventional chemotherapy was efficient.

## Materials and methods

### Inclusion criteria

Patients with metastatic rectal cancer diagnosed between January 2009 and December 2011 were treated at the Department of Oncology at the University hospital of Linköping. The time points (January 2009–December 2011) were chosen since bevacizumab (Avastin) at that time was included as a neoadjuvant therapy to the conventional chemotherapy.

### Descriptive data

Descriptive data such as sex, age, time of diagnosis, date of surgery and date of diagnosis were obtained from the Swedish national register for CRC taken from the cancer centre of the Southeast Swedish Health care region. Information about chemotherapy and RT, the intention of the treatment and the side effects were obtained from medical records.

A total of 39 patients were referred to the MDT conference. Two were excluded due to the lack of metastasis and one due to the lack of information because treatment was taking place at another hospital (records were not available to us). Of the remaining 36 patients, 24 were men and 12 were women. The median age was 65 years for men and 67 years for women (range 38–85 years, Table 1). Twenty-two patients, out of 36, were treated with a curative intention, while 14 patients were considered palliative (Table 2). The decision of whether the patient was curable or palliative was made at the MDT conference.

**Table 1:** The distribution of gender and age of patients with rectal cancer

	Age					
	Mean	Standard deviation	Median	Minimum	Maximum	Column *N* (%)
Gender						
Men	65	10	65	38	83	66.7
Women	66	12	67	47	85	33.3
Total	65	10	66	38	85	100.0

**Table 2:** Intention with treatment distributed by gender

	Gender		
	Men	Women	Total
	Count	Count	Count
Intension with treatment			
Curative intension	15	7	22
Palliative	9	5	14
Total	24	12	36

The most common location for metastasis was the liver (*n* = 32) followed by regional lymph nodes (*n* = 28) and lung metastasis (*n* = 11, Table 3). The numbers of metastases in the group of patients with curative and palliative intention are shown in Table 3.

**Table 3:** Number (a) and distribution (b) of metastases in rectal cancer patients with curative and palliative intention with treatment

	Number of metastasis							
	1		2		3 or more		Total	
	Count	Row *N* (%)	Count	Row *N* (%)	Count	Row *N* (%)	Count	Row *N* (%)
a								
Intention with treatment								
Curative intension	4	18.2	8	36.4	10	45.5	22	100.0
Palliative	3	21.4	5	35.7	6	42.9	14	100.0
Total	7	19.4	13	36.1	16	44.4	36	100.0

	Liver	Lung	Lymph nodes	Other	Total number of patients
b					
Intention with treatment					
Curative intension	20	7	18	7	22
Palliative	12	4	10	6	14
Total	32	11	28	13	36

### Treatment

The chemotherapy for patients treated with a curative intention consisted of capecitabine (Xeloda) in combination with either oxaliplatin (Eloxatin) or irinotecan (Campto) and, if possible, an addition of bevacizumab (Avastin). The chemotherapy was followed by preoperative RT and surgery.

The RT in the curative group consisted of short or long course. The short-course RT was given with 5 Gy in five fractions during a week, a total of 25 Gy. Surgery was then performed without any delay. The long-course RT was given concomitant with capecitabine (Xeloda) with 1.8 Gy in 28 fractions during 6 weeks, a total of 50.4. Surgery was then performed about 5 weeks after completion of RT. Palliative RT is very individual based on the patients’ need of palliation. We did not find any similar treatment patterns for the palliative patients; therefore, the RT data for these patients will not be further discussed.

Patients with low rectal cancers located 0–6 cm from the anal verge or locally advanced tumours underwent abdominoperineal excision (APE). Tumours located 6 cm cranial from the anal verge were treated with total mesorectal excision (TME) with anastomoses or Hartmann’s procedure as appropriate.

Liver surgery was performed at the same time as the primary rectal cancer surgery (synchronously). Other types of metastatic surgery were performed either before or after resection of the primary tumours.

After surgery, the treatment could vary depending on the outcome of the treatment and some patients received palliative treatment. The palliative chemotherapy consisted of the same agents as the curative treatment but with an addition of antibodies against the epidermal growth factor receptor (EGFR) called cetuximab (Erbitux) or panitumumab (Vectibix).

### Ethical approval

The study protocol was approved by the ethical committee in Linköping according to the World Medical Association’s Declaration of Helsinki 1964 and the Amendment of Tokyo in 1975.

### Statistics

The *χ* ^2^ test was used to evaluate whether there was a difference in intention with treatment of men and women as well as between older and younger patients (70 years of age or older and <70 years of age). The Kaplan–Meier method was used to present the differences in overall survival between curative and palliative patients, between men and women as well as between older and younger patients. A *p* value <0.05 was considered statistically significant.

## Results

### Survival

**Fig. 1:**
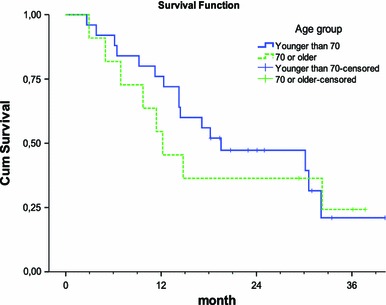
Kaplan–Meier estimates of cumulative survival in patients based on age. Median survival time in patients younger than 70 years was 20 months, and for patients aged 70 years or older, it was 12 months

The overall survival was studied in 36 patients with metastatic rectal cancer treated either with curative or with palliative intention. In all patients, the median survival time was 17 months. In the group of patients older than 70 years, the median survival was 12 months. Five out of 11 patients were considered possible to be cured from start. Patients with an age <70 years had a median survival of 20 months where 17 out of 25 patients were considered possible to be cured (Fig. 1). No significant difference was found between the number of patients possible to be cured in the group of patients older than 70 years and the group of patients younger than 70 years.

**Fig. 2:**
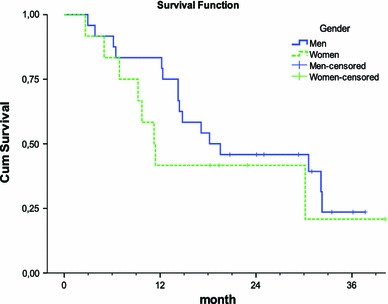
Kaplan–Meier estimates of cumulative survival in patients based on gender. Median survival time for men was 18 months and for women 11 months

Further, the median survival was analysed in men and women separately. The median survival for men was 18 months where 15 out of 24 patients were considered possible to be cured with treatment. The women had a median survival of 11 months where seven out of 12 patients were treated with curative intention (Fig. 2). There was no significant difference between the number of men and women possible to cure.

### Curative intention

**Fig. 3:**
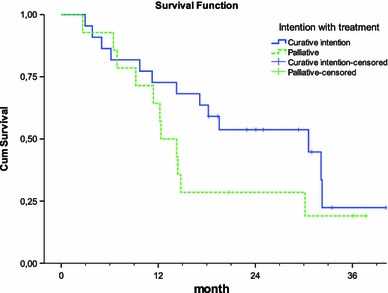
Kaplan–Meier estimates of cumulative survival in curative and palliative intention groups. Median overall survival (*OS*) in patients with curative and palliative intention was 31 and 12 months, respectively

Next, the overall survival for 22 patients with metastatic rectal cancer treated with curative intention was studied. The median overall survival in all patients treated with curative intention was 31 months (Fig. 3).

**Fig. 4:**
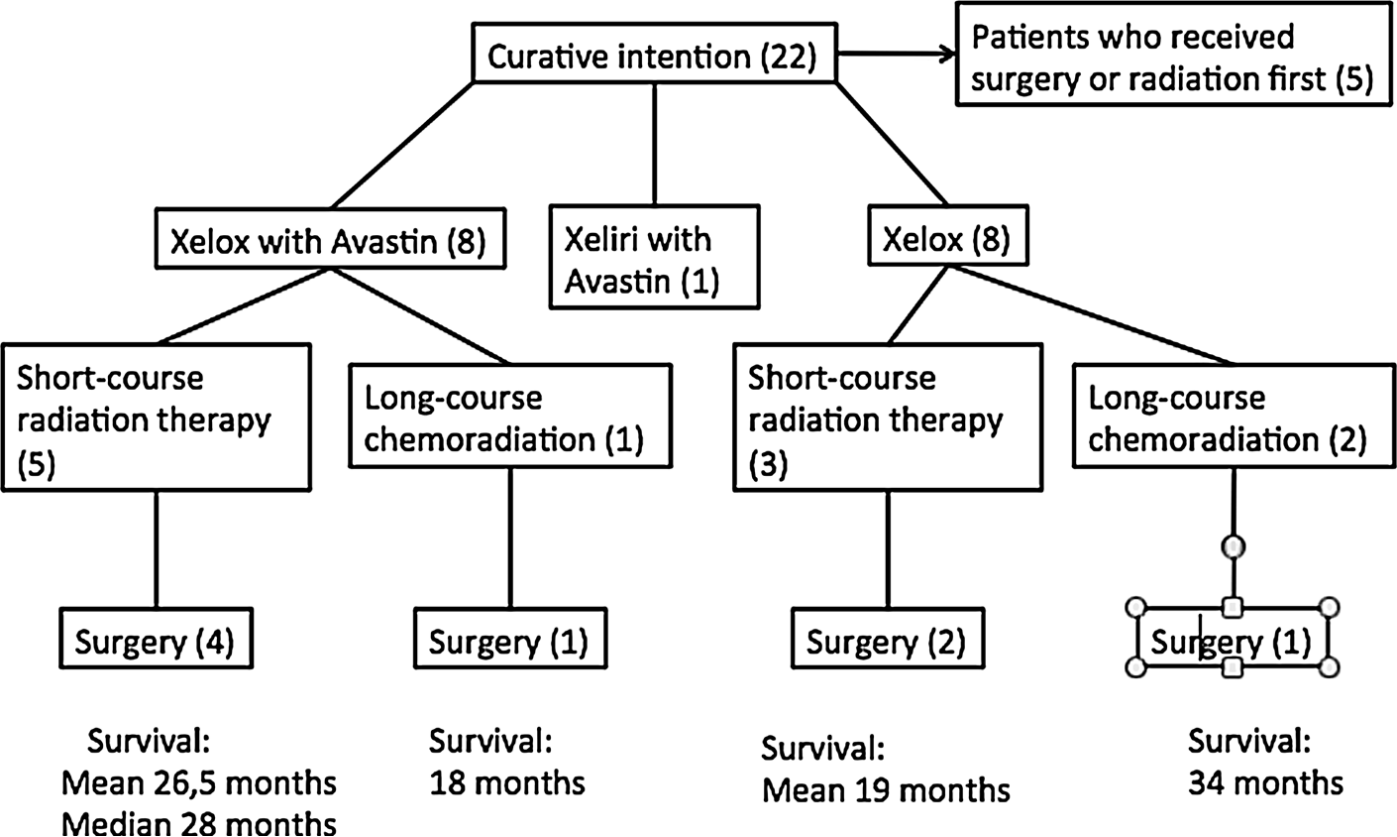
Therapy for the curative intention group. Number of patients receiving each form of therapy *inside the brackets*. Under each arm the survival of the patients who had gone through all the treatments—chemotherapy, radiotherapy (*RT*), and surgery. Some patients become palliative during treatment, counting for the diminishing number in the flow chart

The majority of patients received chemotherapy as their first treatment; however, five out of 22 had surgery or RT as a first treatment (Fig. 4). The chemotherapy was mainly oxaliplatin (Eloxatin) in combination with capecitabine (Xeloda). Eight patients received oxaliplatin (Eloxatin)/capecitabine (Xeloda). Three of these patients received short-course RT, and two of them later had surgery. Two patients who were treated with XELOX received long-course RT, and one of them later had surgery.

Eight patients received the combination of oxaliplatin (Eloxatin)/capecitabine (Xeloda) and bevacizumab (Avastin). Out of eight patients, five were treated with short-course RT and four went on to surgery. One patient who had XELOX and Avastin received long-course chemoradiation and later surgery as shown in Fig. 4.

Four patients (11 %) were considered cured at the end of the study. Two of them received the combination of oxaliplatin (Eloxatin)/capecitabine (Xeloda)/bevacizumab (Avastin) and short-course RT, one patient received oxaliplatin (Eloxatin)/capecitabine (Xeloda) in combination with long-course chemoradiation and one went on to surgery with only preoperative chemotherapy. These four patients had liver metastases or both liver and lymph node metastases at the time of diagnosis.

Eleven patients out of 22 underwent liver and rectal surgery synchronously. Four of the 11 patients later went on to additional liver surgery.

### Palliative intention

**Fig. 5:**
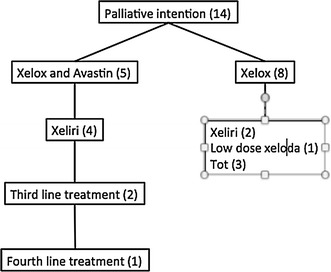
Chemotherapy regimens for palliative patients. Number of patients receiving each form of therapy is shown inside the brackets. Six of the 14 patients received palliative radiation

Further, the overall survival for the 14 patients with metastatic rectal cancer treated with palliative intention was studied. The median overall survival was 12 months (Fig. 3). All patients received chemotherapy except for one (Fig. 5). The first-line palliative treatment was mainly oxaliplatin (Eloxatin)/capecitabine (Xeloda); in addition, five patients received bevacizumab (Avastin). Of the eight patients who received oxaliplatin (Eloxatin)/capecitabine (Xeloda), three got second-line treatment (Fig. 5). Of the five patients with the combination of oxaliplatin (Eloxatin)/capecitabine (Xeloda)/bevacizumab (Avastin), four had second-line treatment, two of them received a third line and one patient received a fourth line of treatment as shown in Fig. 5.

The type of RT treatment varied a lot between the palliative patients since this treatment is used to shrink expansive tumours and relief pain. Six out of 14 palliative patients received RT.

### Side effects

Side effects of the treatments that led to a switch of treatment were found in 16 out of 36 cases (Table 4). The side effects were neuropathy (two patients, 5.6 %), heart attack (one patient, 2.8 %), reduced general condition (two patients, 5.6 %), thrombo-embolic disease (two patients, 5.6 %), gastrointestinal events including diarrhoea (five patients, 13.9 %) and bowel perforation (one patient, 2.8 %). One patient interrupted treatment due to an infection (2.8 %). Five patients received cetuximab (Erbitux), and three of them had an allergic reaction. One of them was serious and life-threatening.

**Table 4:** Side effects for the entire group of the patient material

Side effects	Number of patients
Neuropathy	2
Heart attack	1
Reduced general condition	2
Thromboembolism	2
Gastrointestinal events	5
Bowel perforation	1
Infection	1
Allergy to cetuximab	3
life-threatening	1

Dose-altering side effects are not included here. Gastrointestinal events were mainly diarrhoea, but one of the five patients had the more severe bowel perforation. Cetuximab was given to five patients, and three had an allergic reaction severe enough to change treatment to the fully humanized antibody panitumumab

## Discussion

A problem with research of metastasized rectal cancer is the low number of patients diagnosed with the disease. Therefore, many studies report CRC as an entity [4, 5]. Rectal cancer as an own subgroup has only been explored to a minor extent. The main obstacle for comparing the rectal cancer group with the CRC group is the differences in the preoperative local treatment. In the treatment of rectal cancer patients, not only the RT, chemotherapy and surgery have shown to be of vital importance, but also the optimal timing between the treatments is important. The diagnostic tools and treatments are continuously changed according to new evidence from research. Therefore, it is necessary for hospitals to collaborate in order to perform bigger studies and to be able to compare the treatments. In this study, 39 patients were enrolled where three of the patients were excluded. The patients were rectal cancer patients consecutively referred to the surgical department and received oncological treatment at the University hospital of Linköping. All patients were discussed at the MDT conferences.

In this study, we used a way of dividing the patients into groups based on their possibility to be cured. If a tumour regression was found on the computer tomography (CT) after RT and chemotherapy, the tumours were considered as possible for surgical resection and the patients were considered possible to cure. Dividing the patients into groups based on treatment intention is good since it reflects the reality in patient care and the results are therefore easier to apply in clinical work. Also, side effects are more acceptable if the intention is to cure the patient rather than to give the patient as much time with a good quality of life as possible. It is very important to carefully assess the patient’s chances to be cured before the treatment starts and also during the treatment so that the clinicians insure that the patients receive the best treatment. Many patients in the group with curative intention were later considered palliative, but in this study only their initial clinical status was counted.

The median overall survival for the whole group of patients (*n* = 36) was 17 months, which is in line with the results found in the MRC FOCUS trial [1]. In that study, the overall survival was 15–16 months. The differences between the two studies are that in the MRC FOCUS trial only the patients with inoperable disease were included, while in our study, all the patients intended for surgery were included. Another difference was that in the MRC FOCUS trial only patients with WHO performance status 0–2 were included, and we included all the cases regardless of WHO performance status.

In this study, no significant differences were found in overall survival between patients with curative intention and the patients with palliative intention the first 6 months. The reason for these results might be that during this period most patients are still alive regardless what treatment they have received. After 6 months, a significant difference was found in overall survival between the patients with curative intention and the patients with palliative intention. After around 28 months, the differences in survival between the two groups disappear. Taken together, the results suggest that the patients can live a long time with their disease even though the tumour cannot be removed surgically.

We also showed that 22 out of 36 patients were treated with curative intention, which is the high number of patients compared to how this group of patients was treated 10 years ago. Ten years ago, almost all the patients were considered palliative. It would be interesting in the future to further study which patient would benefit from a more aggressive treatment.

Few studies have analysed the relationship between the treatment with oxaliplatin (Eloxatin)/capecitabine (Xeloda) and bevacizumab (Avastin) in metastatic CRC and overall survival [6, 7]. In this study, we found a median overall survival of 31 months in 22 patients with curative intention treated with the combination of oxaliplatin (Eloxatin)/capecitabine (Xeloda) and, if possible, bevacizumab (Avastin). In another study by Diaz-Rubio et al. [8] on patients with metastatic CRC treated with oxaliplatin (Eloxatin)/capecitabine (Xeloda) and bevacizumab (Avastin), a median survival time of 23.2 months was found. This result shows that more research in this field is necessary in order to improve the preoperative treatment and the patients’ survival.

Our results indicate that 2 months of neoadjuvant chemotherapy followed by RT and then surgery is a feasible treatment. With this approach, one does not risk that the rectum leaves untreated, it is good for both curative and palliative patients, and one has time to perform liver surgery first, if it is clinically indicated.

Side effects are usually described using common terminology criteria of adverse events (CTCAE) from the US department of Health and Human Services [9]. It is a list of common side effects of treatments graded on a scale of 1–5 where one is mild and five is an adverse effect causing death. Many publications, for example, the MRC FOCUS trial, use grade three or four adverse effects to describe their side effects [1]. This was not possible in our study because of insufficient documentation of the severity of side effects in the medical records. Therefore, we used the switch from one chemotherapy regimen to another as a measurement of severe side effects. It was both the doctor’s and the patient’s decisions to end the treatment. Diarrhoea, neuropathy and reduced general condition are well-known side effects to oxaliplatin (Eloxatin)/capecitabine (Xeloda) treatment. Known side effects to bevacizumab (Avastin) include bowel perforation and thrombo-embolic disease. In our study, one bowel perforation was found in patients treated with bevacizumab (Avastin) and two patients suffered from thrombo-embolic disease. Our results show that the risk of side effects by bevacizumab (Avastin) is slightly higher than reported by others. Tebbutt et al. [10] showed that thrombo-embolic events occurred in one of 27 patients (4 %) treated with bevacizumab (Avastin). This difference can be due to a small number of patients in our study or due to the difference in how the adverse events were determined. Although our incidence of side effects is higher than that in other studies, we consider this treatment well tolerable. In the group of patient with palliative intention, cetuximab (Erbitux) was used. One common side effect of cetuximab (Erbitux) treatment is allergic reactions. In this study, we found that three out of five patients with cetuximab (Erbitux) treatment had an allergic reaction to the treatment, and one of the patients even had a life-threatening allergic reaction. This result shows that more than half of the patients receive allergic reaction due to cetuximab (Erbitux) treatment. Although the number of patients is small, we consider this evidence as serious, which must be investigated further.

## Conclusion

We consider the treatment with oxaliplatin (Eloxatin)/capecitabine (Xeloda) in combination with bevacizumab (Avastin) followed by RT as a well-tolerable and good preoperative treatment in patients with metastatic rectal cancer. Eleven per cent of the patients with metastatic disease can be cured by neoadjuvant chemotherapy, RT and surgery. The response to chemotherapy after 2-month treatment is a good prognostic sign for which patients can be cured. Long-lasting palliation can also be obtained with this treatment schedule; however, surgery is not indicated for the palliative patient.
